# Programmable Computational RNA Droplets Assembled via Kissing-Loop Interaction

**DOI:** 10.1021/acsnano.3c12161

**Published:** 2024-06-04

**Authors:** Hirotake Udono, Minzhi Fan, Yoko Saito, Hirohisa Ohno, Shin-ichiro M. Nomura, Yoshihiro Shimizu, Hirohide Saito, Masahiro Takinoue

**Affiliations:** † Department of Computer Science, Tokyo Institute of Technology, 4259 Nagatsuta-cho, Midori-ku, Yokohama 226-8501, Japan; ‡ Department of Life Science Frontiers, Center for iPS Cell Research and Application, Kyoto University, Sakyo-ku, Kyoto 606-8507, Japan; § Department of Robotics, Graduate School of Engineering, Tohoku University, Aoba-ku, Sendai, Miyagi 980-8579, Japan; ∥ Laboratory for Cell-Free Protein Synthesis, RIKEN Center for Biosystems Dynamics Research, Suita, Osaka 565-0874, Japan; ⊥ Department of Life Science and Technology, Tokyo Institute of Technology, 4259 Nagatsuta-cho, Midori-ku, Yokohama 226-8501, Japan; # Research Center for Autonomous Systems Materialogy (ASMat), Institute of Innovative Research, Tokyo Institute of Technology, 4259, Nagatsuta-cho, Midori-ku, Yokohama 226-8501, Japan

**Keywords:** RNA nanotechnology, RNA droplets, molecular computing, phase separation, microRNA diagnostics, molecular robot, artificial cell

## Abstract

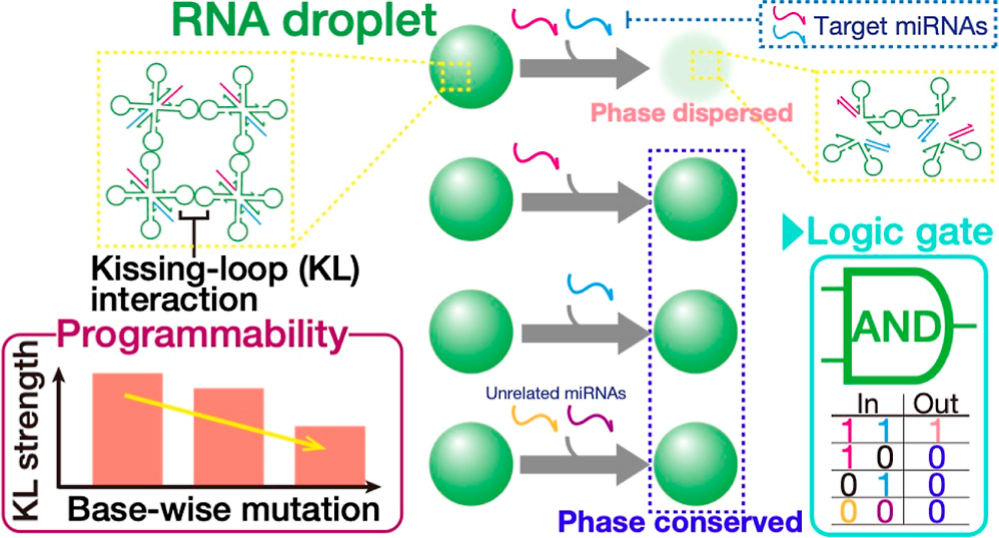

DNA droplets, artificial liquid-like condensates of well-engineered DNA sequences, allow the critical aspects of phase-separated biological condensates to be harnessed programmably, such as molecular sensing and phase-state regulation. In contrast, their RNA-based counterparts remain less explored despite more diverse molecular structures and functions ranging from DNA-like to protein-like features. Here, we design and demonstrate computational RNA droplets capable of two-input AND logic operations. We use a multibranched RNA nanostructure as a building block comprising multiple single-stranded RNAs. Its branches engaged in RNA-specific kissing-loop (KL) interaction enables the self-assembly into a network-like microstructure. Upon two inputs of target miRNAs, the nanostructure is programmed to break up into lower-valency structures that are interconnected in a chain-like manner. We optimize KL sequences adapted from viral sequences by numerically and experimentally studying the base-wise adjustability of the interaction strength. Only upon receiving cognate microRNAs, RNA droplets selectively show a drastic phase-state change from liquid to dispersed states due to dismantling of the network-like microstructure. This demonstration strongly suggests that the multistranded motif design offers a flexible means to bottom-up programming of condensate phase behavior. Unlike submicroscopic RNA-based logic operators, the macroscopic phase change provides a naked-eye-distinguishable readout of molecular sensing. Our computational RNA droplets can be applied to in situ programmable assembly of computational biomolecular devices and artificial cells from transcriptionally derived RNA within biological/artificial cells.

## 1 Introduction

In bioinspired DNA nanotechnology, the sequence recognition capability of DNA enables the self-assembly of complementary single-stranded DNAs (ssDNAs) into well-defined 2D^1,2^ or 3D^3^ nano-/microstructures. The extensive scalability and orthogonality are mediated typically by hybridization between single-stranded overhangs, known as sticky ends (SEs), of rationally designed building blocks. Among others, DNA droplets,^4−14^ liquid-like condensates of well-engineered DNA sequences, have attracted increasing attention as dynamic functional fluid^15^ that bears high programmability in their interactions,^4,16^ physical properties,^9,17^ and phase behaviors.^4,18^ Similar to cellular organelles formed via liquid–liquid phase separation,^19,20^ the membrane-free nature allows DNA droplets to behave as stimuli-responsive dynamic fluid,^15^ such as signal-activated phase separation^4,6^ and enzymatically induced phase-state change.^21^ This programmable dynamic behavior offers a promising alternative to highly sought-after membrane-bound structures^2,22^ in artificial cell studies. Furthermore, DNA droplets have also expanded into molecular computing.^23,24^ AND operations have been performed upon inputs of specific microRNAs (miRNAs) by programming a droplet of cross-linked orthogonal DNAs to phase-separate into distinct phases via target-induced strand displacement reactions.^6^

In parallel, RNA has also provided the reliable basis for the self-assembly of structural^25−27^ and functional^28,29^ analogues.^30−33^ Notably, RNA prefers a more diverse range of self-folded structures and thus unique higher-order structures^31,33,34^ than DNA. One typical example is the kissing loop (KL) interaction between two internally folded single-stranded RNAs (ssRNAs), which allows the self-assembly of folded structures into stable complex structures.^31,35^ The accumulating catalog of RNA structural diversity reported to date describes noncoding RNAs as structural, catalytic, and regulatory^36^ biomolecules^37−39^ beyond the simplistic description of RNA as an information carrier passed on from DNA to protein synthesis. Despite the great potential, it appears that the previous efforts in programmable droplets have paid less attention to RNA as a design material, with an intensive focus placed on SE-assembled DNA droplets.^4,5,7,11,13,16^ The limited repertoire would overshadow the potential growth of programmable droplets into widespread applications that could match the overwhelmingly diverse RNA world. Synthetically derived RNA droplets have been expressed in biological cells. They rely on CAG^40,41^ or CUG^40^ repeat expansions as multivalent interactions mediating liquid–liquid phase separation but still lack design flexibility.

**Figure 1:**
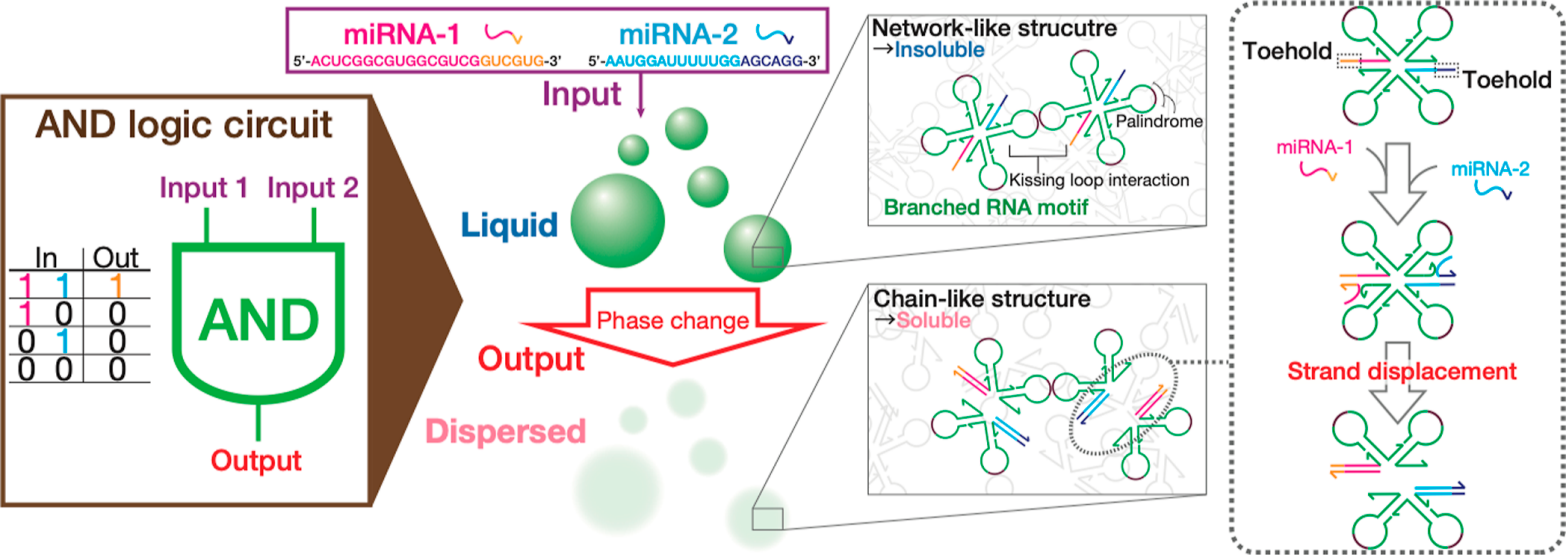
Illustration of computational RNA droplets that carry out AND logic operations. X-shaped motifs (X-motifs) of RNA sequences hybridized in the stem regions self-assemble into the liquid-like condensates via kissing-loop interaction between the two stem-loops. Upon inputs of two specific miRNAs, RNA motifs prefer to split up into two lower-valency structures through strand displacement reactions, resulting in a distinct phase change from liquid to dispersed states.

Here, we present functional RNA droplets capable of performing two-input AND logic operations (Figure 1) that enable the simultaneous detection of multiple miRNA inputs. To maximize the design flexibility, we designed a multistranded RNA motif that can recognize specific miRNAs. The rationally designed RNA motifs self-assemble into a network-like microstructure via KL interaction at the multiple branch ends. Additional two extensions in the motif possess a toehold region designed to bind with a cognate miRNA as a model input molecule, thereby initiating a strand displacement reaction. Upon inputs of two targets, the motif favors a dynamic separation into two substructures, causing a drastic disassembly of the network-like microstructure and hence a liquid-to-dispersed phase-state change. To optimize the design of the sensing motif, we numerically and experimentally study the programmability of the KL interaction strength by engineering the palindromic subsequence. The phase state of RNA condensates was determined by observing the coalescence dynamics and fluorescence recovery after photobleaching (FRAP).

## 2 Results and Discussion

### 2.1 Design of Motifs and Thermostability of Condensates

**Figure 2:**
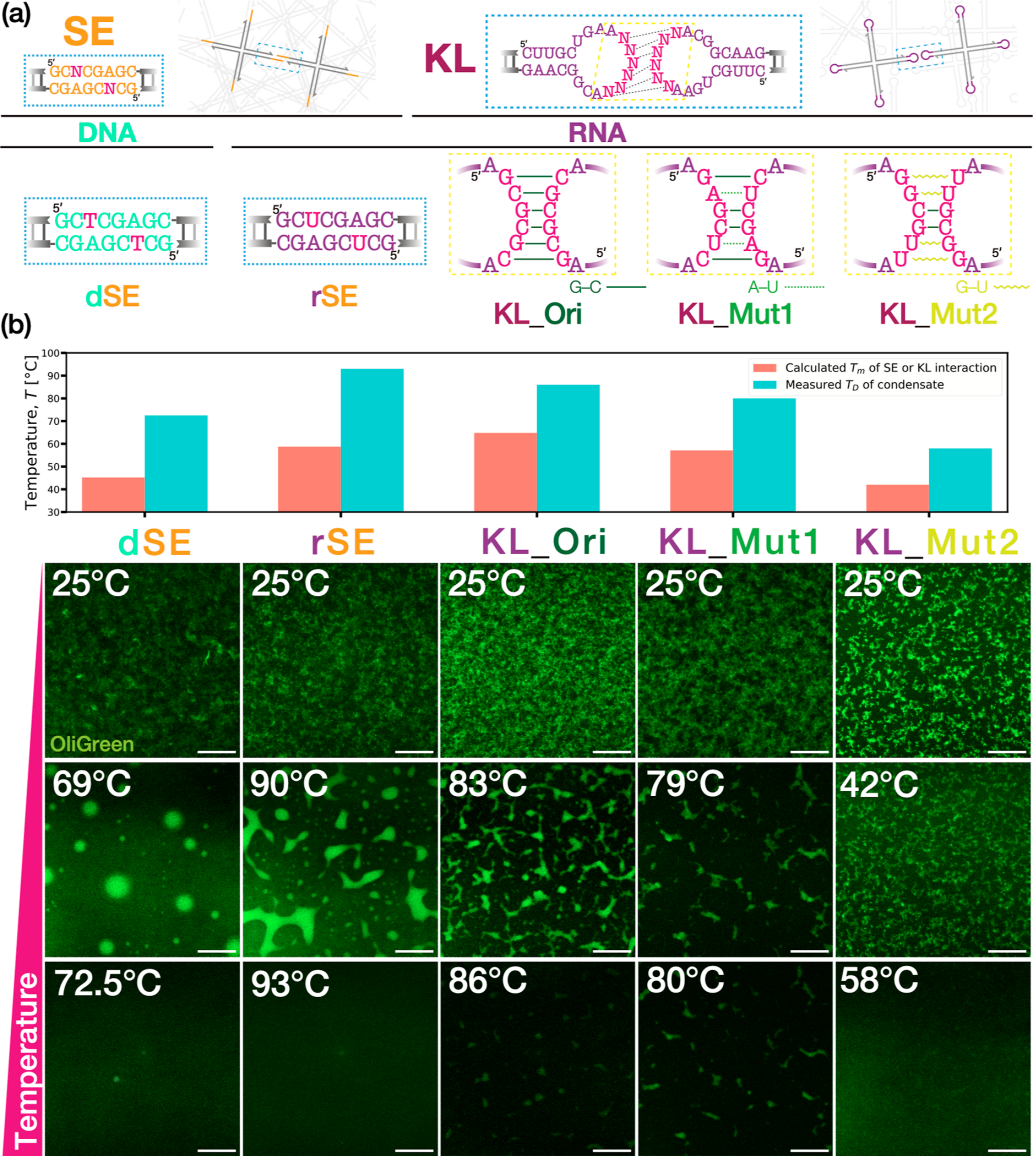
Design features and thermostability of X-motifs using (a) varied materials (DNA or RNA) and interaction mechanisms (SE or KL). (b) Evaluation of SE or KL interaction strength. Melting temperatures *T*_m_ of associated SEs or KLs were numerically calculated by MELTING5^48,49^ and oxDNA,^50,51^ respectively. Dissolving temperatures *T*_D_ of condensates were determined with confocal microscopy observation. DNA/RNA condensates were dyed with OliGreen. Scale bars: 50 μm.

To optimize the motif design for computational RNA condensates, we investigated the thermostability of RNA condensates constructed from RNA nanostructures as a function of different intermotif interactions and varied interaction strength (Figure 2a). Here, we used four-way-branched nanostructures (X-motifs) comprising four ssDNAs or ssRNAs base-paired in the stem regions. In each X-motif, we encoded either of the two interaction mechanisms, SE or KL interaction, at the 5′ end in each component strand. The SE cohesion, which is possible for DNA and RNA, allows X-motifs to self-assemble into network-structured condensates.^3^ We constructed DNA condensates as control using the sequence design (Table S1) well-characterized previously^4^ and RNA condensates based on the RNA analogue (Table S2). Our DNA and RNA sequences that constituted various motifs in this study were designed and validated with the assistance of NUPACK,^42^ a well-known software suite for designing and analyzing nucleic acid-based structures. Note that Figure 2a highlights their SEs as dSE and rSE, respectively, while Tables S1 and S2 refer to the whole motif sequences as dSE-“X” and rSE-“X” with an emphasis on morphological comprehension. This consideration also applies to the other motif terminology mentioned later. The other interaction mechanism was RNA-specific KL interaction between two hairpin loops extending from the branches. Broadly, the KL interaction initiates its process when two stem-loops form a duplex via Watson–Crick base-pairing of their palindromic subsequences, widely shared among the genomic RNAs of retroviruses.^43,44^ This loop–loop interaction then stabilizes the coaxial stacking between the two stems.^38,46^ Here, we designed the palindromic subsequence in the KL region (KL_Ori) as “GCGCGC” (in the 5′ to 3′ direction, which applies hereinafter), which was adapted from that of HIV-1 (LA1) genomic RNA dimerization initiation site.^45,46^ Further, the GC-rich palindrome was tailored to design (1) two-base mutant “KL_Mut1”, where the original two GC base-pairs (bps) were replaced with two AU bps as “GAGCUC” (Table S4), and (2) five-base mutant “KL_Mut2”, where the original four GC bps were converted into four GU bps (a well-known wobble base-pair found in codon–anticodon recognition-pattern degeneracy^47^) as “GGCGUU” (Table S5).

We calculated the melting temperature *T*_m_, a quantitative indicator of the thermostability, in the SE binding for DNA (dSE) and RNA (rSE) and that in the RNA KL interaction for KL_Ori, KL_Mut1, and KL_Mut2 (Figure 2b). The SE binding was treated by MELTING5,^48,49^ free software aimed at predicting the melting temperature of nucleic-acid duplexes, whereas the KL interaction was calculated by oxDNA,^50,51^ another free software suite using coarse-grained models developed for capturing thermodynamics and conformational properties of nucleic acids. The buffer and calculation conditions prescribed in the simulations were invariant (see the Supporting Information for the input conditions). We observed that in the SE binding, the SE cohesion of RNA showed a higher *T*_m_ than that of DNA. This higher stability of rSEs relative to dSEs reiterates the widely reported observations that RNA is typically more stable than DNA,^52^ which has been associated with enhanced base-stacking interaction.^38,53,54^ In the KL interaction, not surprisingly, the oxDNA simulation yielded a decrease in *T*_m_ in the order of KL_Ori > KL_Mu1 > KL_Mut2.

Having numerically predicted the thermostability of the various motifs, we constructed different condensates using the designed X-motifs described above on an isothermal condition instead of a thermal annealing method comprised of multiple thermal gradients. The solutions were incubated for more than 1 h at a fixed temperature of 25 °C (see the Supporting Information for further details). Across all the designed motifs, robust formation of gel structures displaying sponge-like structure was observed at 25 °C (Figure 2b).

Using the fabricated condensates, we carried out melting experiments to determine their dissolving temperature *T*_D_, above which the condensates could be observed to become dispersed with an incremental temperature increase. While *T*_m_ concerns the interaction strength between SEs and KLs, *T*_D_ indicates the thermal stability of the resulting condensate microstructures. *T*_D_ was obtained by incrementally increasing an applied temperature on the samples from room temperature (RT) by a ramp of 5 °C and a much smaller step in the higher temperature range close to *T*_D_. In good correlation with the calculated *T*_m_, a series of the melting experiments yielded a higher *T*_D_ in the RNA condensates of rSE-ended X-motifs than the DNA analog by 20 °C. We also observed a decrease of *T*_D_ in the order of KL_Ori > KL_Mu1 > KL_Mut2. In the middle temperature range, we obtained liquid-like states in the SE-assembled condensates at >60 °C for dSE and >80 °C for rSE. The droplet-like shape of dSE-assembled DNA condensates was reminiscent of thermally annealed DNA droplets fabricated using the same motif design.^4^ The rSE-assembled RNA condensates showed marked wetting on the glass plate, indicating a liquid state. In other motifs, no distinct droplet-like objects were observed. For example, the KL_Mut2-assembled RNA condensates underwent no significant rounding-up until complete dispersion beyond 50 °C. Additionally, the SE-assembled condensates of DNA and RNA exhibited more round morphologies with larger sizes. In contrast, the KL-assembled RNA condensates appeared more rugged with smaller sizes. This morphological difference seemingly indicates the time scales in the underlying mechanisms for the intermotif interaction. The SE-based assembly only requires Watson–Crick base-pairing, while the KL-based assembly additionally undergoes the coaxial stacking between the stem-loops. This longer time scale of the KL-based assembly might affect the condensate fluidity and growth.

The melting experiments suggested KL_Mut2 as a fit-for-purpose KL for designing the sensing X-motif RNA that can operate at moderate temperatures. Strong intermotif interaction can discourage motif reshuffling, leading to decreased fluidity of the condensates. Another motivation for this preference was that KL interaction is unique to RNA. DNA droplets to date have largely been assembled via SE hybridization as multivalency interaction.^4,6,7^ The functionalization ability of KL-assembled condensates that is crucial to sophisticated use in various environments including biological/artificial cells and microreactors remained uncertain. An increased repertoire of motif interactions apart from the SE interaction could potentially inspire widespread applications of our RNA condensates in biological/artificial cells.

### 2.2 AND-Operating RNA Droplets Selectively Sensing Two MiRNAs

**Figure 3:**
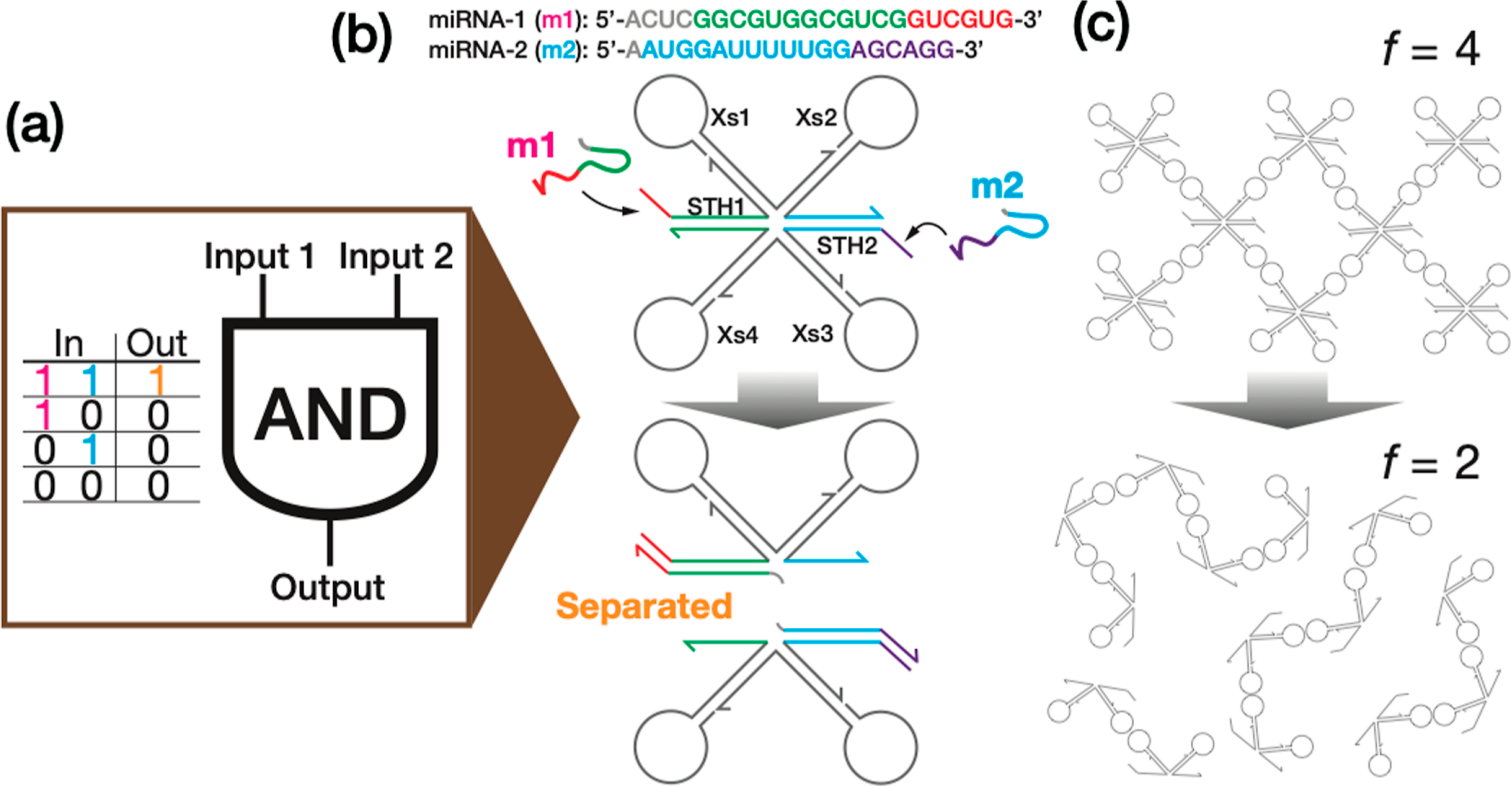
Schematics of (a) AND logic operations using programmable phase behavior of RNA condensate. It is self-assembled by KL interaction between (b) multistranded RNA motifs capable of molecular recognition (termed KL_Mut2-Xs in Table S6). The motifs selectively bind with two miRNAs at STH1 and STH2, each including a toehold. Inputs of the targets mediate strand displacement reactions and thus an irreversible splitting of the quadrivalent motif into two divalent substructures. The motif targets hsa-miR-1307-3p (miRNA-1, “m1”) and hsa-miR-1204 (miRNA-2, “m2”) as model input molecules, which are used for detecting early stage breast cancer.^55^ (c) The resulting conformational change in the condensate. An output of 1 is accompanied by a drastic structural rearrangement of RNA condensates from network-like (insoluble) to chain-like (soluble) structures. *f* denotes the valency number of the RNA motif.

Next, we designed an RNA motif capable of selectively sensing targeted miRNAs based on the KL determined as described above (Table S6). Here, the AND logic was chosen as a model Boolean logic gate, which returns an output of 1 only when two inputs of (1, 1) are entered (Figure 3a). Figure 3b illustrates the designed sensing motif modeled as the two-input AND logic operator and two miRNAs as target input molecules. The miRNAs, m1 and m2, respectively, were referenced from a set of four miRNAs previously reported as an effective biomarker for detecting early stage breast cancer (Table S7).^55^ The designed six-way-branched RNA motif (Table S6) consists of (1) four KL-ended junctions (Xs1–Xs4) and (2) two double-stranded extensions terminating in a toehold (STH1 and STH2). This toehold selectively binds with the cognate part of the target strand and mediates the subsequent strand displacement reaction. To enhance the RNA strand displacement reactions, the substrate toehold was placed at the 5′ end of STH1 and STH2 because the 5′ end toehold can speed up the reaction rate due to RNA’s A-form helix structure.^56,57^ Upon inputs of m1 and m2, i.e., inputs of (1, 1), the X-motif undergoes an irreversible separation into two substructures through the co-occurring strand displacement reactions, which corresponds to the output of 1. At the macroscopic level, this motif restructuring translates into a sizable conformational rearrangement (Figure 3c). The initial-state four-KL motifs favor the self-assembly into a network-like structure in the form of condensate. This robust structure withstands the osmotic pressure from the surrounding buffer and thus behaves as an insoluble dense phase. In contrast, the resulting two-KL substructures prefer to assume chain-like conformation when self-assembled. The decomposed conformation cannot resist the osmotic pressure and thus behaves as a soluble dispersed phase. In other words, the inputs of the targeted miRNAs convert into a drastic phase-state change from liquid to dispersed states through a dynamic reduction of motif valency *f* from *f* = 4 to *f* = 2.

**Figure 4:**
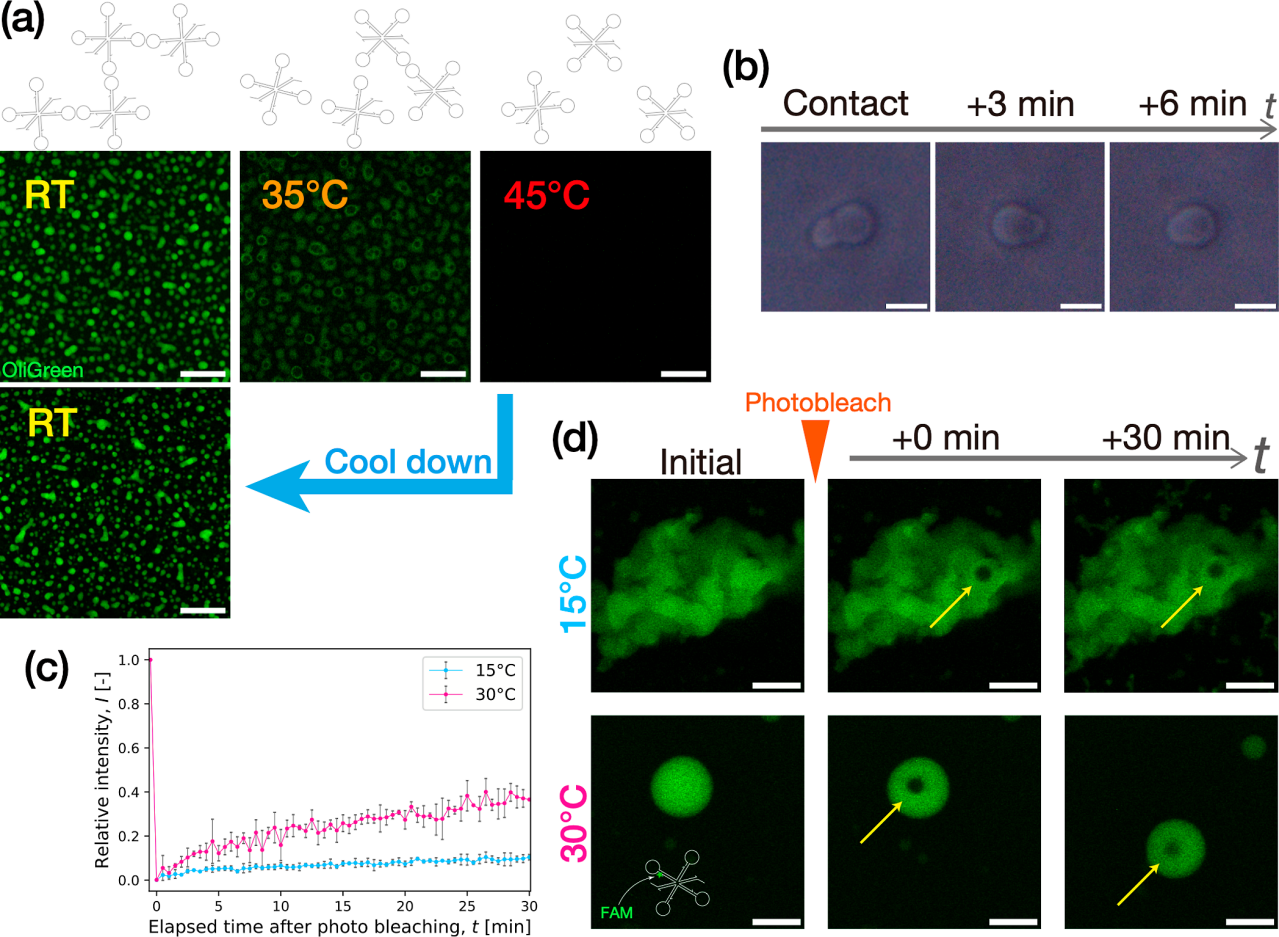
Physical properties of RNA condensates assembled from the sensing RNA motif (Table S6). (a) Thermoreversible phase-state change of the condensates (dyed with OliGreen) captured by confocal microscopy. (b) Coalescence dynamics of RNA condensate particles observed with phase-contrast imaging at 30 °C. (c) Fluorescence recovery profile in FRAP experiments for quantifying the molecular diffusivity in RNA condensates at 15 and 30 °C. Intensity values were normalized by those at *t* = 0 s (see the Supporting Information). *n* = 3. Error bar: SD (d) Representative time-lapse images of photobleached RNA condensates over 30 min. Yellow arrows mark the bleached regions. The motif was labeled with FAM (Supporting Information). Scale bars: (a) 50, (b) 5, and (d) 20 μm.

Before demonstrating the AND logic operations, we experimentally studied the properties of the condensates assembled from the sensing X-motifs. In Figure 4a, we conducted a melting experiment to confirm the thermoreversibility of the condensates by applying a temperature range from RT to 45 °C. Initially, the fabricated RNA condensates assumed a broadly spherical shape, indicating a liquid-like state. With increasing temperature, the condensates showed rounding-up beyond 30 °C, followed by porous formation on the surface at 35 °C. When heated up to 45 °C, the condensates became dispersed completely. Upon cooling down to RT by switching off the heater, we observed a reassembly of the almost spherically shaped condensate, suggesting the thermoreversibility of the condensate. We further captured dynamic coalescence between two particles with time-lapse imaging using phase-contrast (PC) microscopy of the condensates at 30 °C (Figure 4b). Within 6 min of a contact, they showed a fusion into one entity, strongly suggesting that they were in a liquid state. In addition, we conducted FRAP to quantify the molecular diffusivity in the condensates of sensing X-motif. Figure 4c gives a plot of the fluorescence recovery over 30 min after photobleaching. To cancel out the effects from global fluorescence decay and an unwanted drift of the condensate particles of interest, we also quantified the intensity of a region of interest relative to that of a nonbleached region as control (see the Supporting Information for detailed description). We observed a markedly higher fluorescence recovery at 30 °C than at 15 °C. Taken together, these results revealed that the designed sensing motif (KL_Mut2-Xs) could form RNA droplets at 30 °C.

**Figure 5:**
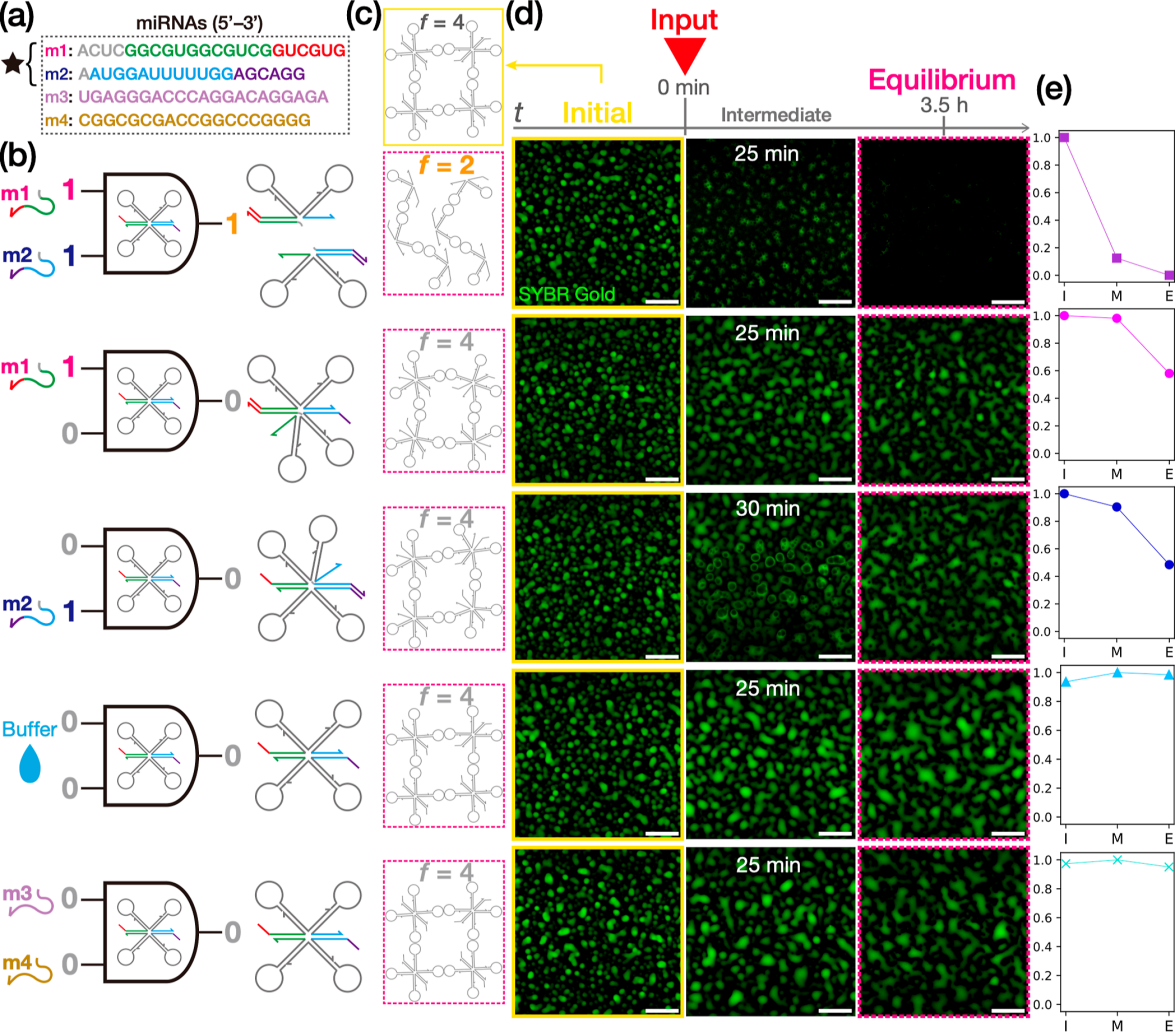
AND logic operations performed by computational RNA droplets. (a) A set of four miRNAs used as model input molecules.^55^ (b) Schematics of different inputs and subsequent motif restructuring of KL_Mut2-Xs (Table S6). (c) Motif conformation in the initial (yellow box) and equilibrium (magenta box) states. In the output of 1, the initial state undergoes a significant rearrangement from network-like (*f* = 4) to chain-like (*f* = 2) structures accompanied by a macroscopic phase-state change; otherwise, the phase state remains invariant due to the conserved motif valency. (d) Confocal microscopy imaging of the RNA droplets (dyed with SYBR Gold) in the initial (yellow box), intermediate, and equilibrium (magenta box) states for varying inputs at 30 °C. Scale bars: 50 μm. (e) Occupancy of bright pixels in a visualized area for different input conditions as an indicator of condensation level in the initial (“I”), intermediate (“M”), and equilibrium (“E”) states (see the Supporting Information for the detail). Data points are normalized by the initial-state occupancy of bright pixels.

Finally, we demonstrate AND logic operations using the RNA droplets, liquid-state condensates of KL_Mut2-X. Besides m1 and m2 as model target molecules (Figure 5a), we considered the aqueous buffer used in this study as control (Figure 5b) and two unrelated miRNAs (m3 and m4), which were also referenced from the biomarker set mentioned above (Figure 5a). Figure 5d presents time-lapse images of the condensates at the initial, intermediate, and equilibrium states for varied inputs. We observed that inputs of both m1 and m2 made a drastic conversion of the initial dense liquid state into a dispersed state over a time period of 3.5 h, as indicated by a significant decrease in the brightness of the visualized area (Figure 5e). In the intermediate state, the RNA droplets showed boiling-like pore formation across the droplets (see Movie S1), indicating nonequilibrium motif restructuring induced by the strand displacement reactions at STH1, 2. This distinct phase-state change strongly suggests that the quadrivalent X-motif experienced a dynamic decrease in valency from *f* = 4 to *f* = 2 (Figure 5c), leading to a global disassembly of the initial network-like conformation.

In an input of either m1 or m2, the RNA droplets maintained the initial dense phase 3.5 h after the input (see also Movies S2 and S3), with a slight decrease in the brightness of the visualized area (Figure 5e). In the single-input reactions, the sensing X-motif allows only one strand displacement reaction, with the valency being conserved throughout the process (*f* = 4), as depicted in Figure 5b. Thus, the initial network-like conformation does not yield to macroscopic disassembly (Figure 5c), although the structural flexibility might increase (Figure 5b). This increased flexibility is prone to the osmotic pressure from the surrounding phase, as indicated by transient pore formation in the middle of the reaction for the input of m2 (Figure 5d and Movie S3). In the inputs of m1 and/or m2 described above, we double-checked the samples after a one-day incubation at RT. In the inputs of both m1 and m2, the dissolved phase state did not yield unwanted recondensation, indicating that the network disassembly was conserved well. In the input of either m1 or m2, the final dense phase remained invariant (Figure S9). These observations firmly suggest that the motif restructuring and resulting phase state were thermally irreversible.

Furthermore, we added the aqueous buffer as a control and unrelated miRNAs, m3 and m4, to RNA droplets to confirm that no unspecific reactions would occur. The initial phase state showed no significant change (Figure 5d,e and Movies S4 and S5), suggesting that the computational RNA droplets can function in a sequence-specific manner.

**Figure 6:**
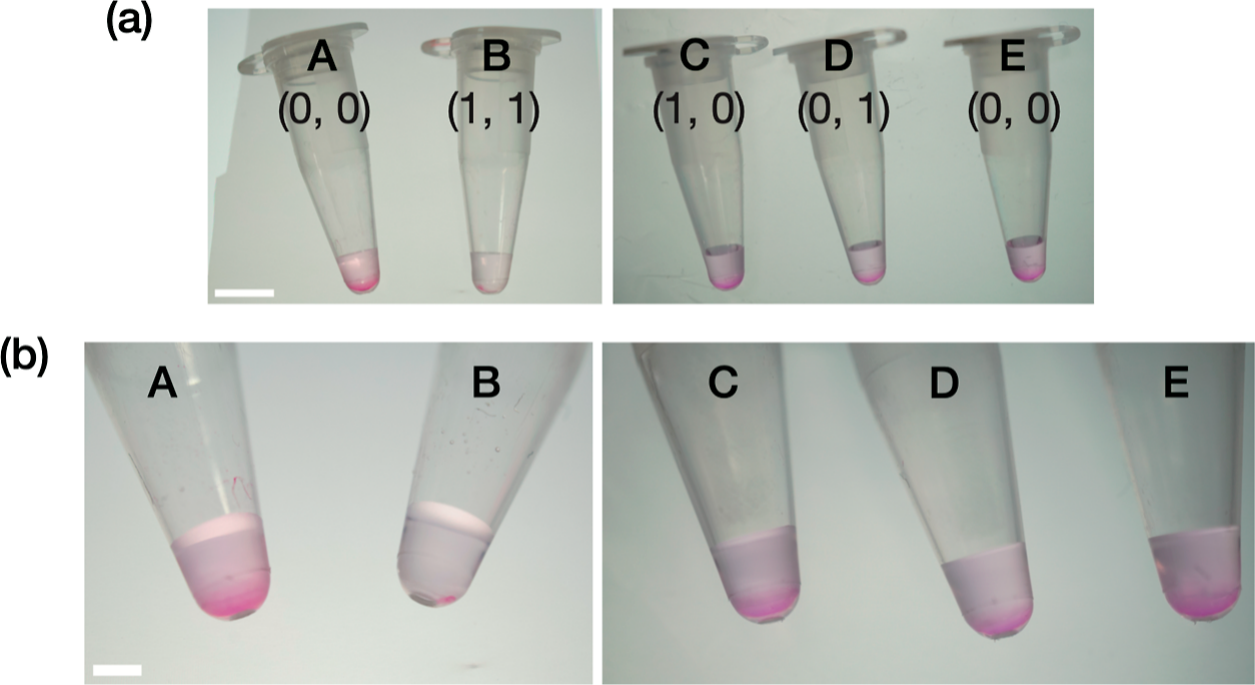
Nonfluorescent naked-eye detection of an AND-gate operation by the computational RNA droplets. (a) Zoomed-out and (b) zoomed-in view of sampling tubes containing MeGP-stained RNA condensate upon the addition of (A,E) the buffer labeled as (0, 0), (B) m1 and m2 as (1, 1), (C) m1 only as (1, 0), and (D) m2 only as (0, 1). Sample volumes: ∼18 μL. Scale bars: (a) 5 and (b) 2 mm.

In the preceding demonstrations, the phase-state changes of fluorophore-labeled RNA condensates were detected using confocal microscopy, a costly method that might hamper their widespread use. A naked-eye-visible detection method of the molecular-level miRNA recognition has the potential to provide facile and robust diagnostic tools available in various contexts. To demonstrate such possibilities, we show a naked-eye detection method of AND operations of the computational RNA condensate system without using confocal microscopy or fluorescence. Upon inputs of m1 and m2, methyl green-pyronin (MeGP), a classical and inexpensive histological nonfluorescent dye that stains DNA and RNA in different colors,^58^ was applied on a computational RNA condensate solution (see the Supporting Information for the procedures). As shown in Figure 6, a two-input-induced AND operation and subsequent phase-state change exhibited only very few stained pellets in a tube, whereas a significant quantity of naked-eye-visible magenta-colored pellets was observed otherwise. This facile and robust fluorescence-free detection method relying on no costly setup would suggest the potential of our RNA droplet device as a powerful diagnostic tool available in various biomedical fields.

In summary, our RNA droplets can perform AND logic operations using programmable phase behavior. The component sensing motif was designed to comprise six ssRNAs hybridized in the stem regions. This multistranded nature gave us beneficial flexibility in designing the motif restructuring as the basis of programmable phase behavior. The specialized roles among multiple strands could integrate two opposing machineries into one motif, where one facilitates the self-assembly of the building blocks into network-like structures, and the other can abolish the network when activated upon inputs of (1, 1). This multistrand design strategy gave us significant flexibility in the bottom-up programming of phase behavior. Another advantage was that the computational process entailed a distinct phase-state change from liquid to dispersed states, which could serve as an easy-to-observe readout of the computational process.

The underlying mechanism of AND operations can be extended to other Boolean logic operations. For example, an OR operation can be programmed using a three-way branched motif, where three KL domains are hybridized with the branched stem comprising multiple strands. A toehold protrudes from the stem region placed upstream of the KL domain. Initially, the trivalent building blocks self-assemble into a space-spanning network-like microstructure as shown by the four-branched motifs. Upon a single input of a specific target, the resulting strand displacement severs the KL domain, thereby reducing the valency from *f* = 3 to *f* = 2. Consequently, the initial space-spanning network-like structure would yield a drastic phase-state change from gel/liquid to dispersed states, corresponding to an OR operation. Furthermore, although being the simplest form of logic operations, RNA-based AND logic computing could be extended to simultaneous parallel processing^59,60^ by designing various sensing orthogonal motifs that function cooperatively.

Note that the breast-cancer biomarkers highlighted herein were not cherry-picked as fit-for-purpose biomarkers based on sweeping tests of many biomarkers. Their previous use as targets in AND-operating DNA droplets^6^ reported by our group allowed us to design and demonstrate the current RNA motif that targets the ready-to-use biomarkers without additional cost. The molecular recognition capabilities of the computational RNA droplet system are enabled by RNA sequence programmability. Thus, our design concept of programming the phase-state change is sequence-independent, and not limited to the current biomarkers, although the motif should be redesigned to target other biomarkers selectively. Finally, it should also be reminded that the RNA condensate system was not designed to form specifically patterned droplets throughout the computational processes. In principle, a single droplet could carry out an AND-gate operation as long as it is observable.

## 3 Conclusions

In this study, we designed and demonstrated computational RNA droplets capable of AND logic operations. Our multistranded motif design enabled the programmable phase-state change through target-induced motif restructuring. We also made a comprehensive parametric study using varied materials (DNA and RNA) and interaction mechanisms (SE and KL). The several-base mutations in the palindromic subsequence of the KL region could fine-tune the thermostability of the resulting RNA condensates, in good correlation with the numerical predictions, which allowed us to optimize the sensing motif design. The RNA condensates assembled from the designed sensing motif were studied experimentally for the thermoreversibility and fluidity, showing that the condensates at 30 °C were in a liquid state. Our computational enzyme-free RNA droplets based on multistrand sensing motifs should provide engineers and scientists with significant flexibility and simplicity in programming phase behavior. Our computational RNA droplets, furnished with high flexibility and detectability, will promise their conversion into far-reaching applications ranging from diagnostic devices for detecting various diseases to functional synthetic cells. They would also be applied to molecular computation devices in living cells by coupling with an intracellular transcription system.

## 4 Methods

Detailed methods and protocols for the experiments, numerical simulations, sequence design, and data analysis are provided in the Supporting Information.

### 4.1 Construction of Condensates

Throughout our experiments, we constructed condensates from the sequences (Tables S1–S6). An equimolar mixture of single-stranded sequences (ssDNAs or ssRNAs) was dissolved into an aqueous buffer within a PCR tube, with the final concentration of 5.0 μM for each ssDNA/RNA, 350 mM for NaCl (>99.5% purity, FUJIFILM Wako Pure Chemical Corp.), and 20 mM for Tris–HCl pH8.0 (UltraPure, Thermo Fisher Scientific). For confocal microscopy observation, the following fluorescent dyes were also added: 1000× Quant-iT OliGreen (ssDNA Reagent, Thermo Fisher Scientific) for studying the SE/KL interaction strength (Figure 2) and 10,000× SYBR Gold (Nucleic Acid Gel Stain, Thermo Fisher Scientific) for demonstrating the AND logic operation (Figure 5). The mixed solutions were incubated at a fixed temperature of 25 °C in a plate thermal cycler (Mastercycler nexus flat, Eppendorf, Hamburg, Germany) for >2 h to allow for the self-assembly of nanostructures into the condensates of DNA or RNA.

### 4.2 Observation Methods

For the fluorescence microscopy observation (Figures 2, 4 and 5) and FRAP experiments (Figure 4), we used a confocal laser scanning microscope (FV1000, Olympus, Tokyo, Japan). For the PC imaging (Figure 4b), the coalescence dynamics of RNA droplets were visualized using fluorescence microscopy (IX71, Olympus). The applied temperatures were regulated by a Peltier heating stage (10021-PE120, Linkam Scientific Instruments Ltd., Surrey, UK) in the confocal microscopy and a thermoplate (TPi-110RX, Tokai Hit Co., Ltd., Fujinomiya, Japan) in the PC imaging.

A sample solution was applied in a silicon sheet cavity as an observation chamber, where a punch-holed silicon rubber sheet of 1.0 mm in thickness (As One, Osaka, Japan) was affixed onto a 3.0 mm × 4.0 mm glass plate with a thickness of 0.13–0.17 mm (no.1, Matsunami Glass Ind., Ltd., Kishiwada, Japan). The glass plate was treated with a BSA (bovine serum albumin, FUJIFILM Wako Pure Chemical Corp.) solution of 5 w/v % BSA and 20 mM Tris–HCl in the experiments using the sensing RNA motif (Figures 4 and 5). Finally, the sample solution was covered with mineral oil (Nacalai Tesque, Inc., Kyoto, Japan) to minimize unwanted evaporation.

## Data Availability

The preprint of the manuscript is available: Hirotake Udono; Minzhi Fan; Yoko Saito; Hirohisa Ohno; Shin-ichiro M. Nomura; Yoshihiro Shimizu; Hirohide Saito; Masahiro Takinoue. Programmable computational RNA droplets assembled via kissing-loop interaction. 2023. ChemRxiv. 10.26434/chemrxiv-2023-qjw7w (October 31, 2023).

## Supporting Information Available

Sequences, design and specificity verification of sequences, native PAGE, construction of condensates, observation methods, sample heating procedures, numerical evaluation of intermotif interaction strength, FRAP, AND logic operations, and fluorescence-free naked-eye detection of AND-gate operation (PDF)
Inputs of m1 and m2 (MP4)
Input of m1 only (MP4)
Input of m2 only (MP4)
Input of buffer (MP4)
Inputs of m3 and m4 (MP4)
