# Supplementary material for: Programmable Computational RNA Droplets Assembled via Kissing-Loop Interaction
Source: ACS Nano. 2024 Jun 4;18(24):15477–86. doi: 10.1021/acsnano.3c12161 (PMC11191694; doi:10.1021/acsnano.3c12161)
Supplement: Supplementary file 1 — nn3c12161_si_001.pdf [file nn3c12161_si_001.pdf]

Programmable Computational RNA Droplets Assembled via Kissing-Loop Interaction

Hirotake Udono,[1] Minzhi Fan,[1] Yoko Saito,[1] Hirohisa Ohno,[2] Shin-ichiro M. Nomura,[3] Yoshihiro Shimizu,[4] Hirohide Saito,[2] Masahiro Takinoue[1,5,6*]

[1] Department of Computer Science, Tokyo Institute of Technology, 4259 Nagatsuta-cho, Midori-ku, Yokohama, 226-8501, Japan

[2] Department of Life Science Frontiers, Center for iPS Cell Research and Application, Kyoto University, Sakyo-ku, Kyoto, 606-8507, Japan

[3] Department of Robotics, Graduate School of Engineering, Tohoku University, Aoba-ku, Sendai, Miyagi, 980-8579, Japan

[4] Laboratory for Cell-Free Protein Synthesis, Center for Biosystems Dynamics Research, RIKEN, Suita, Osaka, 565-0874, Japan

[5] Department of Life Science and Technology, Tokyo Institute of Technology, 4259 Nagatsuta-cho, Midori-ku, Yokohama, 226-8501, Japan

[6] Research Center for Autonomous Systems Materialogy (ASMat), Institute of Innovative Research, Tokyo Institute of Technology, 4259, Nagatsuta-cho, Midori-ku, Yokohama, 226-8501, Japan

*Correspondence to Masahiro Takinoue
E-mail: takinoue@c.titech.ac.jp

<u>This file includes:</u>

- **Supporting Notes, Tables, and Figures**

1. Sequences ···3
2. Design and Specificity Verification of Sequences ·······················5
3. Native PAGE ···7
4. Construction of Condensates···11
5. Observation Methods···11
6. Sample Heating Procedures···11
7. Numerical Evaluation of Inter-Motif Interaction Strength··············12
8. FRAP ··12
9. AND Logic Operations··13
10. Fluorescence-Free Naked-Eye Detection of AND-Gate Operations········14

SI REFERENCES ···15

- **List of Supporting Movies**

Movie S1. Inputs of m1 and m2. A phase change from liquid to dispersed states.

Movie S2. Input of m1 only. No phase change.

Movie S3. Input of m2 only. No phase change. Pore formation due to increased structural flexibility.

Movie S4. Input of buffer. No phase change.

Movie S5. Inputs of m3 and m4. No phase change.

1. Sequences

Below are tables of the DNA/RNA sequences used in this study. Sticky ends (SEs) and kissing loops (KLs) are marked by bold fonts. In each motif, hybridized domains are colored in the same color, while the spacers ('TT' and 'UU') are in gray. The DNA sequences were purchased from Eurofins Genomics (Tokyo, Japan). The RNA sequences and miRNAs were from Sangon Biotech (Shanghai, China) and Hokkaido System Science Co. Ltd. (Sapporo, Japan). Upon arrival, they were dissolved into nuclease-free water (ultrapure DNase/RNase-free distilled water, Thermo Fisher Scientific, MA, US) at a concentration of 100 µM and stored in a –80°C freezer until use.

1.1 X-Motif with SEs

Table S1 X-motif DNA with SEs (dSE-X)[1]

Name	Sequence (5'–3')
dSE-X1	**GCTCGAGC**GCTGGACTAACGGAACGGTTAGTCAGGTATGCCAGCAC
dSE-X2	**GCTCGAGC**GTGCTGGCATACCTGACTTTCGCAAATTTACAGCGCCG
dSE-X3	**GCTCGAGC**CGGCGCTGTAAATTTGCGTTCATCACTTGGGACCATGG
dSE-X4	**GCTCGAGC**CCATGGTCCCAAGTGATGTTCCGTTCCGTTAGTCCAGC

This motif was used to numerically predict the melting temperature of the hybridized SEs (T_m) and experimentally obtain the dissolving temperature of the resulting condensates (T_D) in Figure 2.

Table S2 X-motif RNA with SEs (rSE-X)

Name	Sequence (5'–3')
rSE-X1	**GCUCGAGC**GCUGGACUAACGGAACGGUUAGUCAGGUAUGCCAGCAC
rSE-X2	**GCUCGAGC**GUGCUGGCAUACCUGACUUUCGCAAAUUUACAGCGCCG
rSE-X3	**GCUCGAGC**CGGCGCUGUAAAUUUGCGUUCAUCACUUGGGACCAUGG
rSE-X4	**GCUCGAGC**CCAUGGUCCCAAGUGAUGUUCCGUUCCGUUAGUCCAGC

This motif was used to obtain T_m (SE) and T_D (condensates) in Figure 2.

1.2 X-Motif with KLs

In addition to KL_Ori-X (Table S3), we designed two X-motif RNAs with weaker KL interaction by adapting the self-complementary subsequence in the KL. We replaced two GC base-pairs (bps) with two AUs for KL_Mut1-X and with two GUs (wobble bps) for KL_Mut2-X. In the KL interaction, two KLs form a helix via the Watson–Crick base-pairing in the self-complementary segments (underlined in the Tables), and then establish the tertiary structure via the coaxial stacking between the two loops.

Table S3 X-motif RNA with KLs (KL_Ori-X)

Name	Sequence (5'–3')
KL_Ori-X1	**CUUGCUGAAGCGCGCACGGCAAG**GCUGGACUAACGGAACGGUUAGUCAGGUAUGCCAGCAC
KL_Ori-X2	**CUUGCUGAAGCGCGCACGGCAAG**GUGCUGGCAUACCUGACUUUCGCAAAUUUACAGCGCCG
KL_Ori-X3	**CUUGCUGAAGCGCGCACGGCAAG**CGGCGCUGUAAAUUUGCGUUCAUCACUUGGGACCAUGG
KL_Ori-X4	**CUUGCUGAAGCGCGCACGGCAAG**CCAUGGUCCCAAGUGAUGUUCCGUUCCGUUAGUCCAGC

This motif was used to obtain T_m (KL interaction) and T_D (condensates) in Figure 2.

Table S4 X-motif RNA with KLs (KL_Mut1-X)

Name	Sequence (5'–3')
KL_Mut1-X1	...GAGCUC...
KL_Mut1-X2	...GAGCUC...
KL_Mut1-X3	...GAGCUC...
KL_Mut1-X4	...GAGCUC...

The omitted segments (...) are equal to the non-modified counterparts of KL_Ori-X.

This motif was used to obtain T_m (KL interaction) and T_D (condensates) in Figure 2.

Table S5 X-motif RNA with KLs (KL_Mut2-X)

Name	Sequence (5'–3')
KL_Mut2-X1	...GGCGUU...
KL_Mut2-X2	...GGCGUU...
KL_Mut2-X3	...GGCGUU...
KL_Mut2-X4	...GGCGUU...

The omitted segments (...) are equal to the non-modified counterparts of KL_Ori-X.

This motif was used to obtain T_m (KL interaction) and T_D (condensates) in Figure 2.

1.3 Branched RNA Motif Capable of Sensing Specific MiRNAs

In the sensing RNA motif (Table S6), STH1,2 include a short single-stranded overhang as a toehold (underlined by dashed lines), which prefers to bind with the complementary region of the target miRNAs (m1 and m2, respectively. See Table S7) and initiate the strand displacement reaction. Only in the presence of m1 and m2, the 4-valence sensing RNA motif splits up into two 2-valence motifs. Otherwise, the valence is conserved, although the structural flexibility increases in the input of only m1 or m2. The displaced strand becomes a single-stranded extension protruding from the neighboring branch. The KL regions were adapted from those of KL_Mut2 (Table S5).

The set of miRNAs (Table S7) is the tumor marker for detecting early-stage breast cancer.[2]

In the fluorescence recovery after photobleaching (FRAP) experiments described below, we labeled the sensing RNA motif by replacing 0.5 μM of KL_Mut2-Xs2 (10% of the final concentration) with 0.5 μM FAM-labeled ssDNA (Table S8) instead of adding the staining reagents.

Table S6 Sensing RNA motif with KLs (KL_Mut2-Xs)

Name	Sequence (5'–3')
STH1	CACGACCGACGCCACUUCCGUUCCGUUAGUCCAGC
STH2	CCUGCUCCAAAAAUCUUCGCAAAUUUACAGCGCCG
KL_Mut2-Xs1	CUUGCUGAAGGCGUUACGGCAAGGCUGGACUAACGGAACGGUUAGUCAGGUAUGCCAGCAC
KL_Mut2-Xs2	CUUGCUGAAGGCGUUACGGCAAGGUGCUGGCAUACCUGACUUUGAUUUUUGG
KL_Mut2-Xs3	CUUGCUGAAGGCGUUACGGCAAGCGGCGCUGUAAAUUUGCGUUCAUCACUUGGGACCAUGG
KL_Mut2-Xs4	CUUGCUGAAGGCGUUACGGCAAGCCAUGGUCCCAAGUGAUGUUGUGGCGUCG

This motif was used to construct the computational RNA droplets investigated in Figures 3, 4, and 5.

Table S7 miRNAs[2] (m1,2: target; m3,4: non-target)

Name	Sequence (5'–3')
hsa-miR-1307-3p as 'm1'	ACUCGGCGUGGCGUCGGUCGUG
hsa-miR-1204 as 'm2'	AAUGGAUUUUUGGAGCAGG
hsa-miR-6875-5p as 'm3'	UGAGGGACCCAGGACAGGAGA
hsa-miR-4634 as 'm4'	CGGCGCGACCGGCCCGGGG

These RNA sequences were used as inputs for performing the logic operations in Figure 5.

Table S8 FAM-labeled DNA sequence

Name	Sequence (5'–3')
FAM_dXs2	[FAM]-GTGCTGGCATACCTGACTTTTTTTGG

This fluorophore-labeled sequence was utilized in the FRAP experiments (Figure 4d).

2. Design and Specificity Verification of Sequences

Among the DNA/RNA sequences mentioned above, dSE-X (Table S1) is one of the DNA motifs featured and characterized by our previous paper.[1] rSE-X (Table S2) is the RNA analog of this DNA motif. The KL-ended RNA motifs (Table S3–Table S5) were adapted from rSE-X by replacing the SEs with the KLs including the varied palindromic subsequences. To provide KL_Mut2-Xs (Table S6) with the recognition capability, we added the two extensions including the toehold regions to KL_Mut2-X (Table S5), which were designed to initiate strand displacements with the two target miRNAs, respectively.

To verify the specificity in the structure and input-induced rearrangement of KL_Mut2-Xs, we used NUPACK, a well-known software suite for the design and analysis of nucleic-acid constructs.[3] Herein, since the other RNA motifs share the stem regions with dSE-X well-studied previously with NUPACK,[1] the following description is focused on the generated analysis data for KL_Mut2-Xs.

2.1 Simulated Structure of Six SsRNAs as Components of KL_Mut2-Xs

To simulate the resulting structure from the multiple RNA sequences, we employed a NUPACK 4.0 Python package (Python version: 3.10.12). The model was specified as 'material = RNA', 'celsius = 37', and 'sodium

= 0.35'; the complex consisted of the six ssRNAs (Table S6) at a concentration of 5.0 µM in a tube with the max complex size of 6. The tube analysis predicted the designed structure at approximately 5.0 µM (Figure S1a). To visualize the resulting structure of KL_Mut2-Xs, the six ssRNAs were grouped and concatenated into two strands for ease of calculation. The two sequences were entered in the web-based NUPACK utilities, generating a 6-branched folded structure similar to the designed 6-branch KL_Mut2-Xs (Figure S1b). These simulated results satisfied us that the six ssRNAs would constitute the designed 6-branched KL_Mut2-Xs motif with high fidelity.

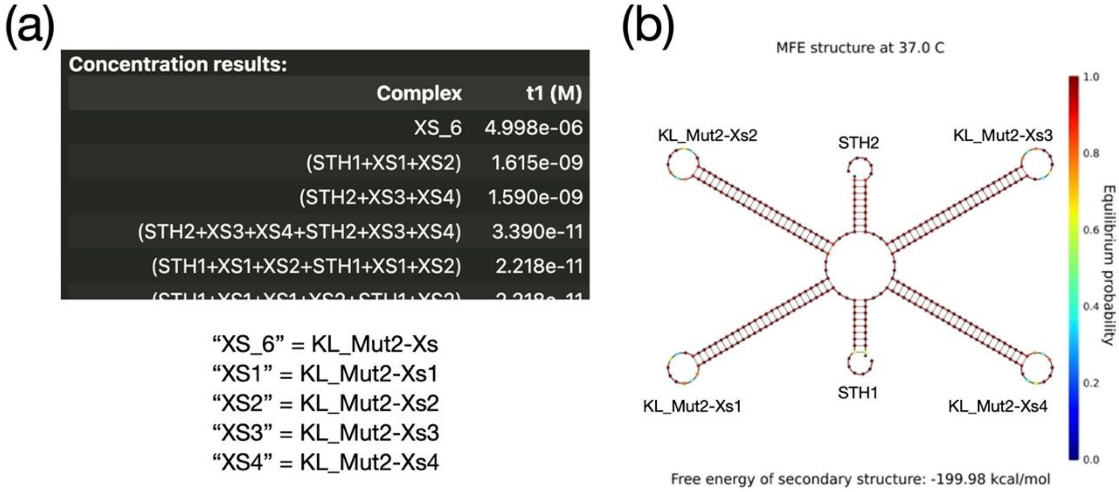

Figure S1 Simulated structure of 5.0 µM six ssRNAs constitute KL_Mut2-Xs (Table S6). (a) Screenshot of possible structures generated by NUPACK 4.0 (Python package). The resulting structures are listed in the order of equilibrium concentration. The top structure corresponds to the designed KL_Mut2-Xs. 37°C, 350 mM NaCl. (b) Folded structure of the six ssRNAs concatenated into two strands predicted by the web-based NUPACK utilities. 37°C, 1 M NaCl.

2.2 Input-Induced Rearrangement of KL_Mut2-Xs

Next, we conducted numerical predictions of the possible rearrangement of KL_Mut2-Xs in the presence of the target miRNAs (m1 and m2) to examine how the resulting strand displacement reactions could affect the motif structure. KL_Mut2-Xs and related miRNAs were entered into the NUPACK 4.0 (Python package) at 5.0 µM and 5.1 µM, respectively, with the same model as the construction verification. It was suggested that upon the addition of both m1 and m2, KL_Mut2-Xs favored a distinct separation into two substructures, each comprising two consecutive KL domains binding with the related miRNAs (Figure S2a). This means that the motif reduces the valency from $f = 4$ to $f = 2$ upon the addition of both m1 and m2. In the presence of m1 only (Figure S2b) or m2 only (Figure S2c), the most likely structure apparently experienced only one strand displacement with no structural separation, and thus conserved the initial valency of $f = 4$. These numerical results suggested that KL_Mut2-Xs would show the strand displacements sequence-specifically and hence the AND-gate operations.

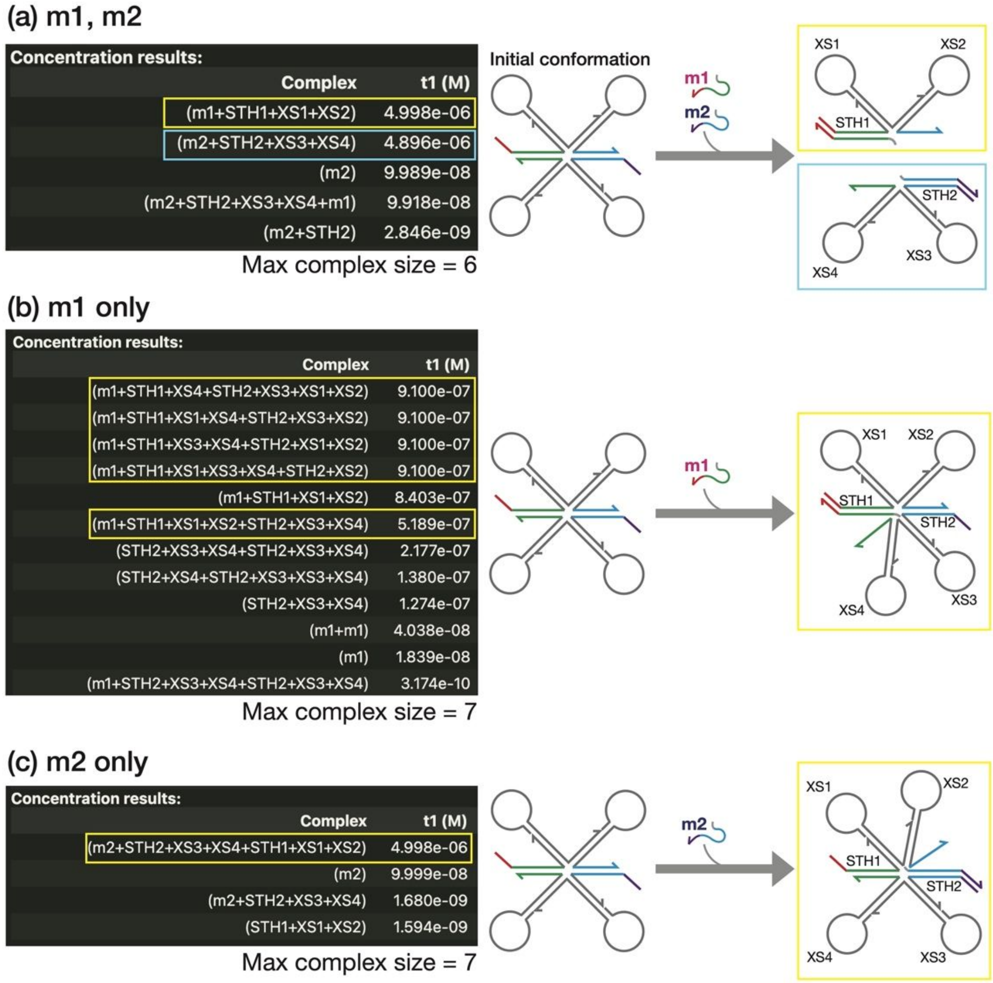

Figure S2 Resulting structures of 5.0 µM KL_Mut2-Xs in the presence of the target miRNAs simulated by NUPACK 4.0 (Python package). 37°C, 350 mM NaCl. Left, screenshots of the predicted structures upon the addition of (a) m1 and m2, (b) m1 only, and (c) m2 only; right, illustrations of the most feasible structures from the generated concentration results. They are marked by yellow and light blue rectangles in the screenshots.

3. Native PAGE

The motifs listed above were analyzed by non-denaturing polyacrylamide gel electrophoresis (native PAGE). A mixture of 5.0 mL 40 w/v%-Acrylamide/Bis Partitioned Solution (29:1, Nakalai Tesque, Kyoto, Japan) and 4.0 mL 5× TBE (Tris-borate EDTA Buffer, Nippon Gene, Toyama, Japan) was topped up to 20 mL with dH_2O in a test tube to prepare an 8 cm × 8 cm gel plate with 10% concentration. After adding 150 µL of 10 w/v% APS (ammonium persulfate, FUJIFILM Wako Pure Chemical Corp.), the tube was gently inverted several times. After adding 3.0 µL TEMED (N, N, N', N'- tetramethylethylenediamine, FUJIFILM Wako Pure Chemical Corp.), the tube was again inverted similarly. For polymerization, the solution was incubated for 60 min in an assembled gel electrophoresis cassette (BE-240G, Bio Craft, Tokyo, Japan).

Next, a 1.0 µL 1:1 mixture of a sample solution and a 2× loading buffer was loaded into each lane. 1.0 µL

10-bp ladder was loaded in the leftmost lane. The 2× loading buffer contained 1.0 w/v% BPB (bromophenol blue, FUJIFILM Wako Pure Chemical Corp.), 10 v/v% glycerol (FUJIFILM Wako Pure Chemical Corp.), and 500 mM EDTA (ethylenediaminetetraacetic acid, Nippon Gene). Afterward, gel electrophoresis was conducted at RT with a PowerPac Basic Power Supply (Bio-Rad Laboratories, CA, US) for 30 min at a constant voltage of 200 V in a cooler box (~4°C). The 10-bp ladder was a lab-fabricated mixture of purified polyT dsDNAs of 10–100 bp. The HPLC-purified polyTs were purchased from Eurofins. Finally, for fluorescence staining, the gel plate was soaked and gently shaken at RT for 5 min in 100 mL 1× TBE containing 0.01 v/v% SYBR Gold. Finally, the gel plate was scanned by a 473-nm excitation laser and imaged by a fluorescence image analyzer (FLA-5100, Fujifilm, Tokyo, Japan).

The native PAGE showed that in each motif, the distinct bands for its component strands disappeared in the rightmost lane for the self-assembled condensates achieved after 2 h incubation.

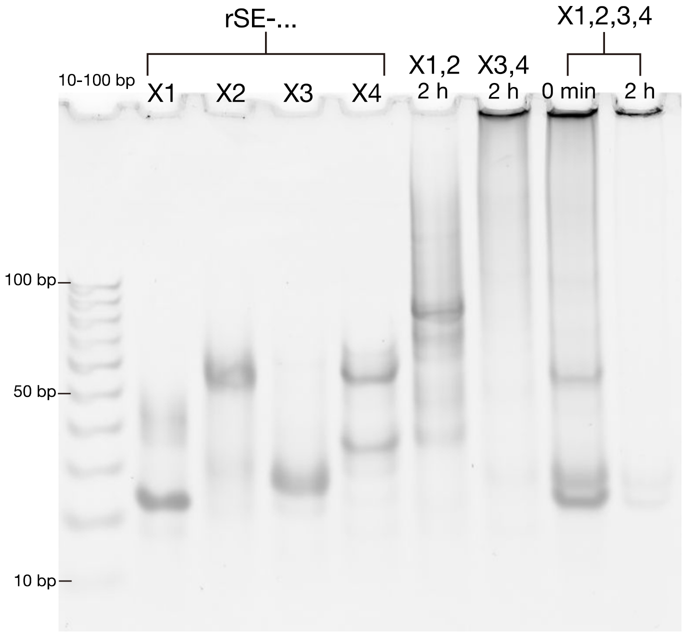

Figure S3 Native Page analysis of rSE-X. The time periods mean an incubation time length.

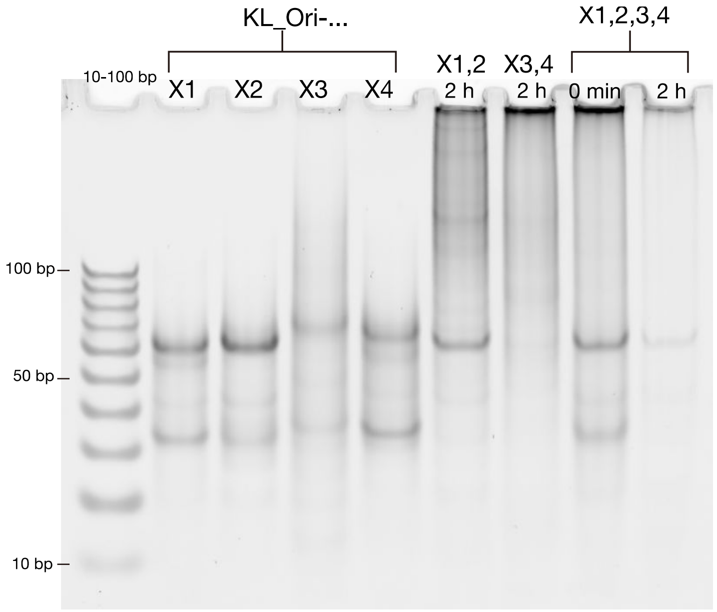

Figure S4 Native Page analysis of KL_Ori-X. The time periods mean an incubation time length.

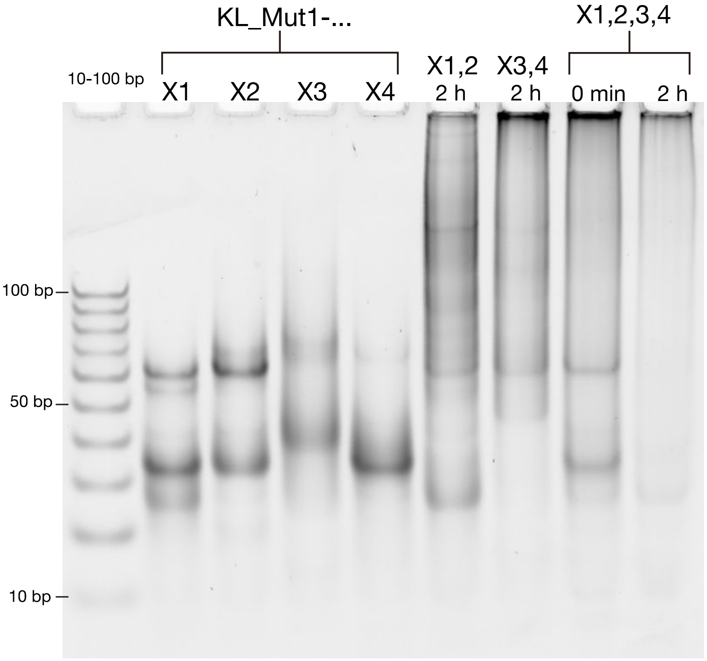

Figure S5 Native Page analysis of KL_Mut1-X. The time periods mean an incubation time length.

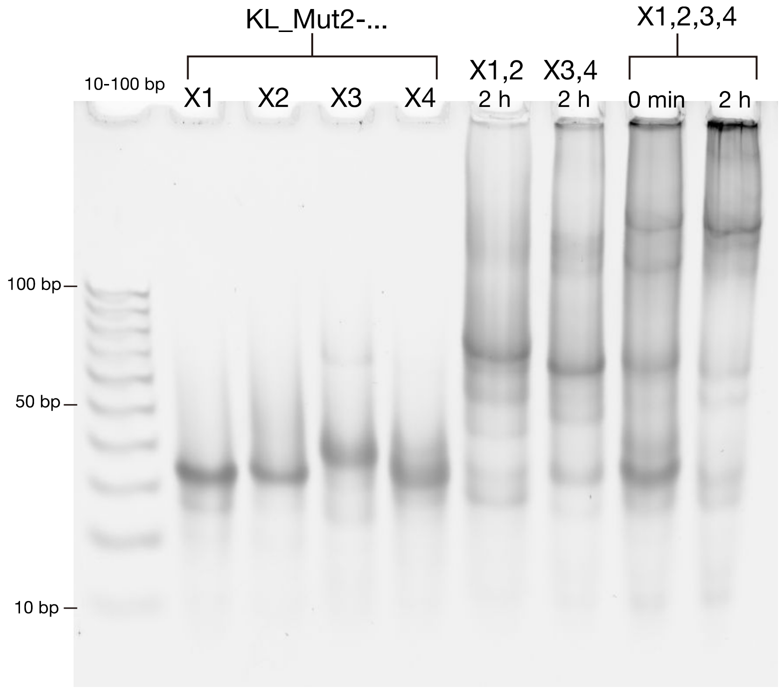

Figure S6 Native Page analysis of KL_Mut2_X. The time periods mean an incubation time length.

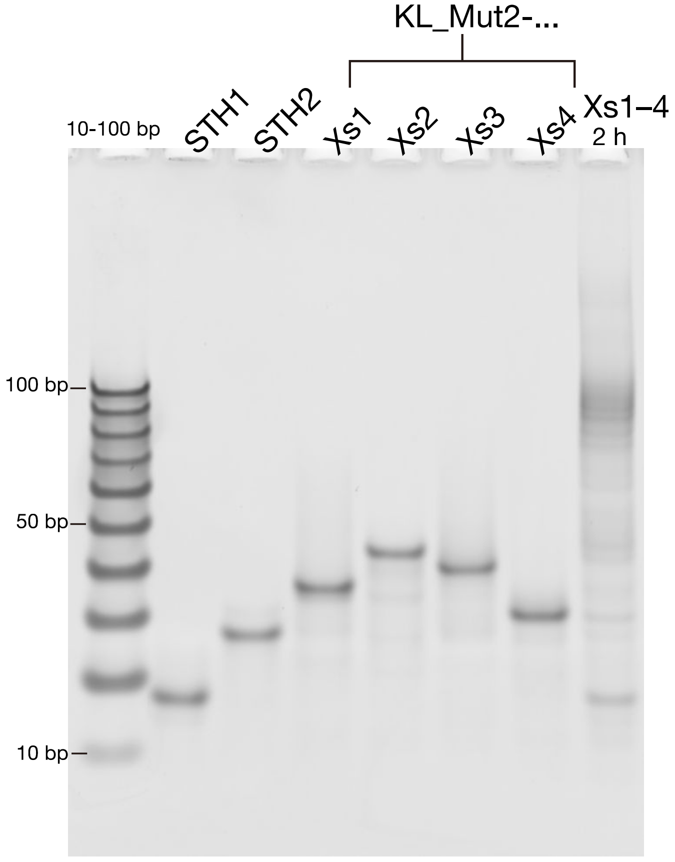

Figure S7 Native Page analysis of KL_Mut2_Xs. The time periods mean an incubation time length.

4. Construction of Condensates

Throughout our experiments, we constructed condensates from the sequences listed above (Table S1–Table S6). An equimolar mixture of single-stranded sequences (ssDNAs or ssRNAs) was dissolved into an aqueous buffer within a PCR tube, with the final concentration of 5.0 µM for each ssDNA/RNA, 350 mM for NaCl (> 99.5% purity, FUJIFILM Wako Pure Chemical Corp.), and 20 mM for Tris-HCl pH8.0 (UltraPure, Thermo Fisher Scientific). For confocal microscopy observation, the following fluorescent dyes were also added: 1000× Quant-iT™ OliGreen (ssDNA Reagent, Thermo Fisher Scientific) for studying the SE/KL interaction strength (Figure 2) and 10000× SYBR™ Gold (Nucleic Acid Gel Stain, Thermo Fisher Scientific) for demonstrating the AND logic operation (Figure 5). The mixed solutions were incubated at a fixed temperature of 25°C in a plate thermal cycler (Mastercycler® nexus flat, Eppendorf, Hamburg, Germany) for >2 h to allow for the self-assembly of nanostructures into the condensates of DNA or RNA.

5. Observation Methods

For the fluorescence microscopy observation (Figures 2, 4, 5) and FRAP experiments (Figure 4), we used a confocal laser scanning microscope (FV1000, Olympus, Tokyo, Japan). For the phase-contrast (PC) imaging (Figure 4b), the coalescence dynamics of RNA droplets were visualized using fluorescence microscopy (IX71, Olympus). The applied temperatures were regulated by a Peltier heating stage (10021-PE120, Linkam Scientific Instruments Ltd., Surrey, UK) in the confocal microscopy and a thermoplate (TPi-110RX, Tokai Hit Co., Ltd., Fujinomiya, Japan) in the PC imaging.

A sample solution was applied in a silicon sheet cavity as an observation chamber, where a punch-holed silicon rubber sheet of 1.0 mm in thickness (As One, Osaka, Japan) was affixed onto a 3.0 mm × 4.0 mm glass plate with a thickness of 0.13–0.17 mm (No.1, Matsunami Glass Ind., Ltd., Kishiwada, Japan). The glass plate was treated with a BSA (bovine serum albumin, FUJIFILM Wako Pure Chemical Corp.) solution of 5 w/v% BSA and 20 mM Tris-HCl in the experiments using the sensing RNA motif (Figure 4, 5). Finally, the sample solution was covered with mineral oil (Nacalai Tesque, Inc., Kyoto, Japan) to minimize unwanted evaporation.

6. Sample Heating Procedures

In the thermostability (Figure 2b) and melting (Figure 4a) experiments, a sample confined in an observation chamber was placed on the thermoplate at room temperature (RT) and underwent the following heating procedure. For the thermostability experiments, the applied temperature was increased with a ramp of 5°C in the lower temperature range and a much smaller step in the upper temperature range close to the condensates T_D. After reaching each temperature studied, the sample was imaged twice at elapsed times of 1 h and 2 h with the confocal microscopy. If the phase of condensates remained, the sample was further heated up to the next

higher temperature. If the sample was observed to dissolve, the applied temperature was recorded as T_D. For the melting experiment, a sample was subjected to a similar heating procedure up to 45°C, where the sample showed complete dispersion.

7. Numerical Evaluation of Inter-Motif Interaction Strength

For numerically evaluating the SE binding strength (Figure 2), we used MELTING5,[4] free software for the prediction of melting temperatures of nucleic acids. In calculating the binding strength of dSE–dSE (Table S1) and rSE–rSE (Table S2), the components of the solutions as described above were entered in the input commands.

For numerically investigating the KL interaction, we employed oxDNA,[5, 6] widely used coarse-grained numerical model for exploring the thermodynamic and structural properties of nucleic acids. The Monte Carlo simulations were performed on the following conditions: NVT ensemble at 30°C, 0.5 M sodium concentration, umbrella sampling with histograms being extrapolated to temperatures ranging from 25°C to 75°C, total time steps of 1.0×10^8, delta translation set as 0.15, and delta rotation as 0.2. Melting temperatures T_m (Figure 2) were obtained using a Python script from the generated histograms.

8. FRAP

In the FRAP experiments (Figure 4), the sensing RNA condensates self-assembled from KL_Mut2-Xs (Table S6) were photobleached to produce a bleached region of ~6 μm (19 pixels) on the surface as a region of interest (ROI). The Peltier stage was set at 15°C and 30°C, at which the condensates would prefer gel and liquid states, respectively. After capturing the time sequence over 30 min, the fluorescence recovery in the ROI was processed and analyzed using Fiji.[7] We noticed that in some cases, the RNA condensates experienced a disturbance-induced drift or slight fluorescence decay. To cancel out these effects on the quantification of the fluorescence recovery, we picked up a non-bleached region as a control in the data analysis process (Figure S8). We then calculated the ratio of the intensities from both regions and rescaled it to a 0–1 range.

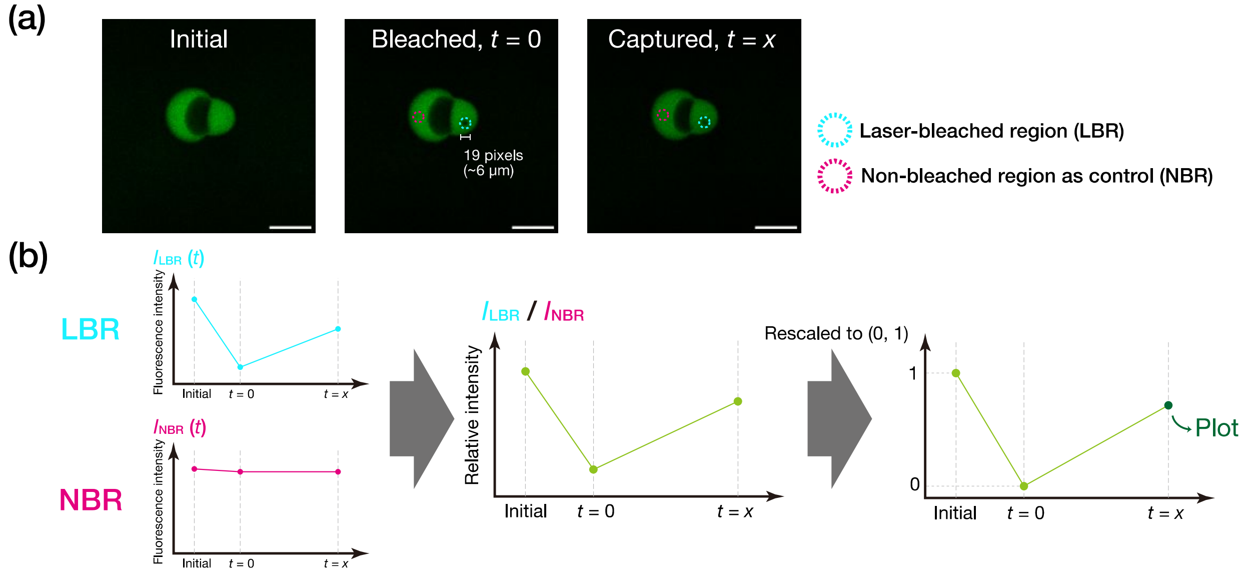

Figure S8 FRAP procedures. (a) Representative confocal microscopy images of photobleached RNA condensates (in a liquid-like state). Scale bars: 50 µm. (b) Description of the data analysis of captured fluorescence recovery.

9. AND Logic Operations

RNA condensates were assembled from KL_Mut2-Xs (Table S6) in a PCR tube at a fixed temperature of 25°C in the plate thermal cycler. Afterward, aqueous buffers containing miRNAs (Table S7) as an input were added, with the final concentration of [KL_Mut2-Xs] = 5.0 µM and [miRNA] = 5.0 µM. Following a two-hour incubation at 25°C, the sensing RNA condensates were visualized with FV1000 on the Peltier heating stage fixed at 30°C.

Figure 5d gives plots of the occupancy of bright pixels in a confocal microscopy image to quantify the condensation level of computing RNA condensates at the initial, intermediate, and equilibrium states. Pixels were judged to be 'bright' when their intensity values were higher than a specific threshold value of 8 in the 16-bit grayscale. The occupancy was calculated by dividing the number of the bright pixels with the entire area of the obtained image.

In the main manuscript, we stated that inputs of both m1 and m2 created a drastic phase-state change in the RNA condensates from liquid to dispersed states, whereas the input of either m1 or m2 led to no significant phase change. To support this finding, we ascertained that the resulting phase states would not return to the initial state after an extended time period. In Figure S9, we observed the samples used in the input experiments after an overnight incubation at RT. In the inputs of m1 and m2, the dispersed state was conserved well; in the inputs of either m1 or m2, the dense phases were also preserved well, with a slight recovery in the fluorescence. This is firm evidence that the motif restructuring and resulting macroscopic conformations at 30°C suggested in Figure 5 was not a transient but a thermally irreversible process.

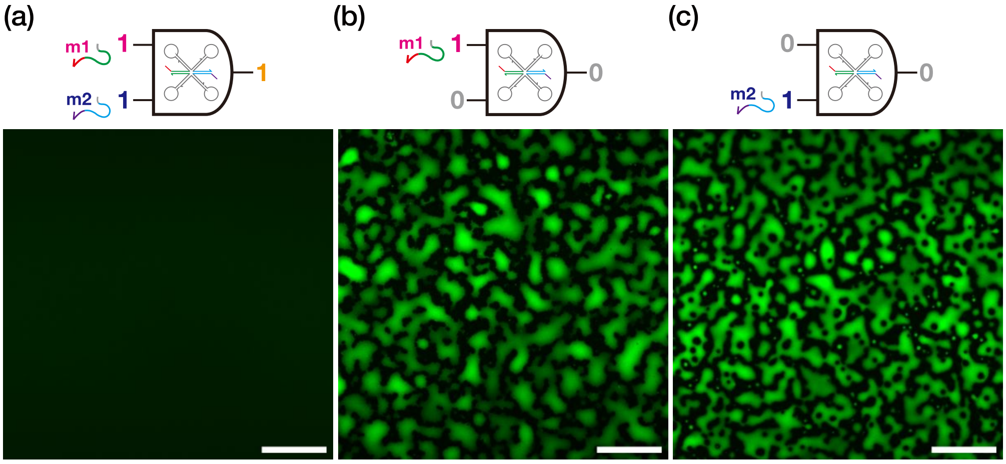

Figure S9 Thermal irreversibility of the computational process of RNA droplets. Confocal microscopy images of the samples observed one day after use in the input experiments. Inputs of (a) both m1 and m2, (b) only m1, and (c) only m2. Stainign dye, SYBR Gold. Scale bars: 50 µm.

10. Fluorescence-Free Naked-Eye Detection of AND-Gate Operations

KL_Mut2-Xs condensates were fabricated in the aqueous buffer at 5.0 µM as described above. As shown in Figure 6, the RNA condensate solutions in PCR tubes were mixed with 20 µM target miRNAs for inputs of m1 and m2 as labeled (1, 1), m1 only as (1, 0), and m2 only as (0, 1) at the final concentration of 3.6 µM KL_Mut2-Xs and 5.6 µM input miRNAs, and the buffer as (0, 0), respectively. After a 90 min incubation at RT, the resulting 13.84 µL sample solutions were mixed with 1.38 µL methyl green-pyronin (Nakalai Tesque, 10 × diluted with the buffer) and incubated at RT for 5 min. Subsequently, the tubes were centrifuged by a centrifuge (CT15RE, HITACHI, Tokyo, Japan) at 15,000 rpm, 25°C for 10 min to obtain pellets at the tube bottoms. To remove redundant stain in the tubes, 13 µL supernatants were exchanged with an equal amount of the buffer solutions. Upon an additional cycle of the centrifuge and buffer exchange, the tubes were imaged using a digital single-lens reflex camera (α6400, SONY, Tokyo, Japan) with a macro lens (SEL30M35, SONY). For background illumination, the tubes were placed on an iPad (MYFQ2J/A, Apple, Inc., CA, US) displaying the bright white screen of a flashlight app.

SI REFERENCES

1. Sato Y, Sakamoto T, Takinoue M. Sequence-based engineering of dynamic functions of micrometer-sized DNA droplets. *Sci Adv*. 2020;6(23):eaba3471.
2. Shimomura A, Shiino S, Kawauchi J, *et al.* Novel combination of serum microRNA for detecting breast cancer in the early stage. *Cancer Sci*. 2016;107(3):326–334.
3. Zadeh JN, Steenberg CD, Bois JS, *et al.* NUPACK: Analysis and design of nucleic acid systems. *J Comput Chem*. 2011;32(1):170–173.
4. Dumousseau M, Rodriguez N, Juty N, Le Novère N. MELTING, a flexible platform to predict the melting temperatures of nucleic acids. *BMC Bioinformatics*. 2012;13:101.
5. Ouldridge TE, Louis AA, Doye JPK. Structural, mechanical, and thermodynamic properties of a coarse-grained DNA model. *J Chem Phys*. 2011;134(8):085101.
6. Snodin BEK, Randisi F, Mosayebi M, *et al.* Introducing improved structural properties and salt dependence into a coarse-grained model of DNA. *J Chem Phys*. 2015;142(23):234901.
7. Schindelin J, Arganda-Carreras I, Frise E, *et al.* Fiji: an open-source platform for biological-image analysis. *Nat Methods*. 2012;9(7):676–682.
